# Persistence of a T Cell Infiltrate in Human Ganglia Years After Herpes Zoster and During Post-herpetic Neuralgia

**DOI:** 10.3389/fmicb.2019.02117

**Published:** 2019-09-11

**Authors:** Jeremy P. Sutherland, Megan Steain, Michael E. Buckland, Michael Rodriguez, Anthony L. Cunningham, Barry Slobedman, Allison Abendroth

**Affiliations:** ^1^Emergency Department, Westmead Hospital, The University of Sydney, Sydney, NSW, Australia; ^2^Discipline of Infectious Diseases and Immunology, The University of Sydney, Sydney, NSW, Australia; ^3^Department of Neuropathology, Royal Prince Alfred Hospital, The University of Sydney, Sydney, NSW, Australia; ^4^Department of Pathology, The University of Sydney, Sydney, NSW, Australia; ^5^Centre for Virus Research, Westmead Institute for Medical Research, Westmead, NSW, Australia

**Keywords:** herpes zoster (HZ), varicella-zoster virus, ganglia, post-herpetic neuralgia (PHN), immune cell

## Abstract

Varicella-zoster virus (VZV) is a human herpesvirus which causes varicella (chicken pox) during primary infection, establishes latency in sensory ganglia, and can reactivate from this site to cause herpes zoster (HZ) (shingles). A major complication of HZ is a severe and often debilitating pain called post-herpetic neuralgia (PHN) which persists long after the resolution of the HZ-associated rash. The underlying cause of PHN is not known, although it has been postulated that it may be a consequence of immune cell mediated damage. However, the nature of virus-immune cell interactions within ganglia during PHN is unknown. We obtained rare formalin fixed paraffin embedded sections cut from surgically excised ganglia from a PHN-affected patient years following HZ rash resolution. VZV DNA was readily detected by qPCR and regions of immune infiltration were detected by hematoxylin and eosin staining. Immunostaining using a range of antibodies against immune cell subsets revealed an immune cell response comprising of CD4^+^ and CD8^+^ T cells and CD20^+^ B cells. This study explores the immune cell repertoire present in ganglia during PHN and provides evidence for an ongoing immune cell inflammation years after HZ.

## Introduction

Varicella zoster virus (VZV) is a highly species specific human herpesviruses responsible for two clinically distinct human diseases. Primary infection results in varicella (chickenpox), during which the virus establishes latency within neurons in the sensory ganglia (Gilden et al., 1983, 1987; Hyman et al., 1983; Lungu et al., 1995; Kennedy et al., 1998; LaGuardia et al., 1999; Levin et al., 2003; Wang et al., 2005). Latency is maintained throughout the life of the host. During latency there is cessation of viral replication and no production of progeny virions (Depledge et al., 2018). Herpes zoster (HZ) also known as shingles is the result of reactivation from latency, and may be followed by a severe neuropathic pain called post-herpetic neuralgia (PHN). PHN is defined as pain lasting longer than 90 days following the onset of the HZ rash (Watson et al., 1988a; Schmader, 2007; Steiner et al., 2007). PHN affects approximately 10% of all and 30% of elderly patients following HZ. In 30–50% of such patients it persists for more than 1 year, and in some for many years and often has significant negative affects on their quality of life (Coplan et al., 2004; Dworkin et al., 2007, 2008; Kawai et al., 2014).

The underlying pathology and pathogenesis of PHN is unknown, and investigations into PHN are complicated by the rare nature of relevant clinical samples and the non-availability of a suitable animal model. PHN may result from damage to the sensorineural pain pathway during HZ, resulting in malfunctioning of sensory neurons causing the transmission of pain signals (Bennett, 1994; Steiner et al., 2007). An alternate hypothesis is the presence of ongoing viral replication and/or immunopathology within the sensory ganglia resulting from immune infiltration against viral antigens (Steiner et al., 2007). The development of PHN is predicted by the severity of acute HZ pain [reviewed in Kawai et al. (2015)].

Previous studies of human ganglia from PHN-affected individuals have been limited to basic histological examination of rare post-mortem material from PHN-affected patients. To date, there have only been three studies, which have noted infiltrations of inflammatory cells and general cell loss within the dorsal root ganglia (DRG) which innervate the painful area (Smith, 1978; Watson et al., 1988b, 1991a). Our laboratory has developed immunohistochemical and immunofluorescent based assays to investigate immune cell subsets in ganglia months after contracting HZ and during active VZV reactivation. Our previous study of ganglia obtained 1–5 months after contracting HZ revealed a robust immune cell infiltrate is still present following the disappearance of the rash and is comprised largely of non-cytolytic (granzyme B-negative) T cells and macrophages (Gowrishankar et al., 2010). More recently, we examined several ganglia from a patient who died with a HZ rash and demonstrated that CD4^+^ and cytolytic CD8^+^ T cells may play an important role in the ganglia during active HZ (Steain et al., 2014). However, to date there have been no detailed studies of ganglia obtained from an individual experiencing clinically defined, prolonged PHN (years after resolution of the HZ rash).

As a result of our very limited understanding of the ongoing pathology of PHN, treatment options are very limited. There is a range of pharmacological, interventional and surgical options available. However, in most cases the pain can often be severe and intractable (Wu and Raja, 2008; Chen et al., 2014) and some patients continue to suffer until death despite the successful introduction of pregabalin, a substantial advance (Snedecor et al., 2014). However, until the mechanism(s) underlying the cause of PHN are better understood, treatment and prevention strategies will remain limited, and patients will continue to develop and suffer from the devastating effects of PHN.

The aim of this study was to investigate VZV antigen expression and nucleic acid levels within ganglion samples from a PHN-affected patient (years following rash resolution) and a HZ-affected patient (presence of HZ rash). These rare clinical samples were also used to define for the first time the nature of the immune cell subsets present within human ganglia years after HZ rash resolution in an individual suffering from PHN.

## Results

### Description of Cases and Histological Examination of Human Ganglia During Post-herpetic Neuralgia

One of the greatest obstacles to the study of PHN and HZ is the difficulty in obtaining appropriate human ganglia samples. For this study we obtained very rare surgically excised PHN-affected ganglia, as well as post-mortem ganglia from a patient suffering HZ at the time of death, and two control cases consisting of ganglia from individuals without any evidence of VZV disease.

The PHN-affected patient was a 59 year old female who was diagnosed with acute myeloid leukemia, and underwent a bone marrow transplant. During recovery the patient experienced HZ in the right thoracic region (T9–T12), and following the resolution of the rash experienced prolonged pain (>5 years) around the same anatomical site. After exhausting all conservative treatment options a ganglionectomy was performed in an attempt to control the pain, with right thoracic T11 ganglion removed. Nine months later, a second ganglionectomy was performed, with right thoracic T10 removed. Surgery was performed when the patient was 60 years of age. Both of the right T10 and right T11 ganglia (termed PHN1 and PHN2, respectively) were utilized in this study.

The HZ patient was a 93 year old male with frontotemporal dementia who died due to aspiration pneumonia. This patient developed a HZ rash approximately 17 days prior to death. At autopsy there was a hemorrhagic vesicular and ulcerated rash in a left lumbar dermatome. The ganglion innervating the site of the rash (left lumbar L2) was collected, and noted to be inflamed.

The first control patient thoracic ganglion (CON1) was from a 73 year old female who died due to metastatic adenocarcinoma. The second control patient ganglion (CON2) was from a 73 year old female who died due to a head injury. Medical records indicate that these patients experienced no clinical VZV reactivation prior to their death.

**FIGURE 1:**
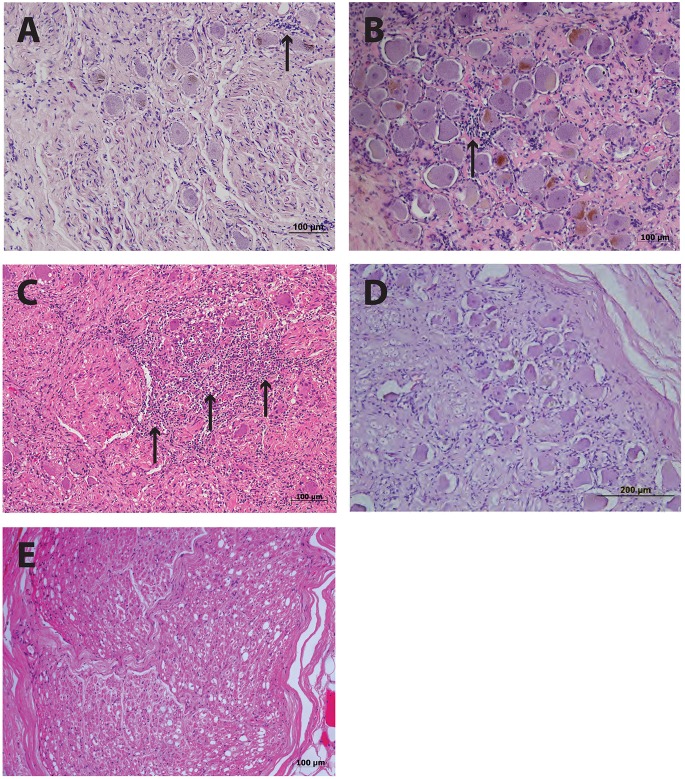
Histology of human ganglia during post-herpetic neuralgia (PHN) and herpes zoster (HZ). Representative images of hematoxylin and eosin staining ganglion sections from PHN ganglia sample 1 **(A)** and sample 2 **(B)** showing infiltrates of small round immune-like cells (arrows). Ganglion sections from a case of active HZ **(C)**, and control ganglia sample 1 **(D)** and 2 **(E)**.

Histological examination of ganglia samples utilized a hematoxylin and eosin (HE) stain on 5 μm formal fixed paraffin embedded (FFPE) sections with representative images shown in Figure 1. This staining revealed focal infiltrations of small immune-like cells within both the PHN1 and PHN2 ganglia samples (Figures 1A,B). There was a greater infiltration of small round immune-like cells spread throughout the HZ ganglion sample (Figure 1C). In the sections examined we observed no significant areas of neuronal cell loss. Intact neuronal profiles surrounded by satellite cells were observed in ganglia from the two control patients but there were no apparent infiltration of immune-like cells (Figures 1D,E).

### Ganglia Samples Were Infected With VZV, but Not HSV-1

Viable DNA was successfully isolated from all ganglion samples. The presence of VZV DNA was confirmed in all PHN and HZ-affected material via PCR, yet the closely related virus HSV-1 was not detected (data not shown). Thus, the immune cell infiltration observed in these ganglionic samples was not a consequence of HSV-1 infection.

To further investigate VZV DNA load a real-time PCR approach for VZV open reading frame 28 (ORF28) was employed. Nucleic acids were extracted from sections and the VZV DNA ganglia load calculated. Sufficient DNA was obtained from the PHN2, HZ and CON2 samples only. Human albumin is present in tissue as one copy per cell, and provides a standard against which the copy number of VZV DNA was calculated. There were 2.95 × 10^5^ copies of VZV DNA per 10^5^ cells in PHN2 and 4.27 × 10^4^ copies of VZV DNA per 10^5^ cells in HZ. No VZV DNA was detected in CON2. This result demonstrates that VZV DNA was readily detectable in ganglia of a PHN-affected patient years after the resolution of their HZ rash and may provide evidence of an ongoing virological process during PHN.

### Immunohistochemical Investigation of VZV Antigen Expression in Human Ganglia Samples

Detection of VZV antigen expression in FFPE material has proven to be problematic (Zerboni et al., 2012). Thus we utilized our well-established immunohistochemical (IHC) assay with an antibody against VZV immediate early protein 63 (IE63), to assess VZV antigen expression within ganglionic sections.

**FIGURE 2:**
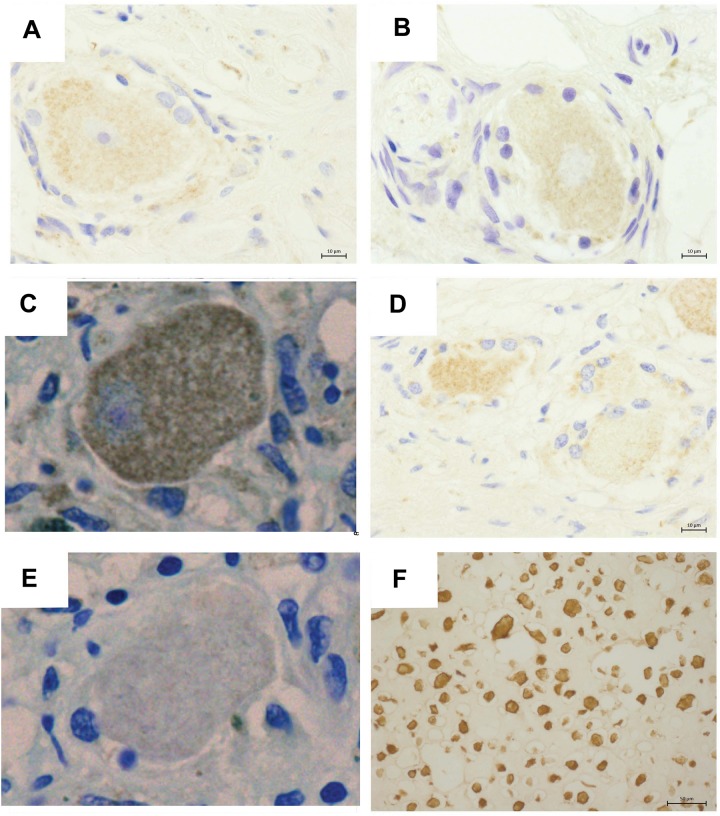
Immunohistochemical detection of VZV antigens in human ganglia from patients experiencing herpes zoster. Representative images of ganglionic sections from PHN1 **(A)** and PHN2 **(B)**, HZ **(C)**, and CON1 **(D)**, as well as a positive control section of infected human fibroblasts stained **(F)** with an anti-VZV IE63 specific antibody. Isotype control antibodies were also applied to a HZ ganglion section **(E)**. Bound primary antibodies were visualized using DAB substrate, and sections were counterstained with Azure B.

Varicella zoster virus IE63 staining was not detected in PHN1 and PHN2 affected ganglia (Figures 2A,B). In contrast, VZV IE63 antigen expression was observed at low levels within regions of the HZ ganglia from the site of reactivation (Figure 2C). No staining was observed on control ganglionic samples (Figure 2D) or ganglionic sections incubated with isotype control antibodies (Figure 2E). FFPE human neurons typically show background staining with antibodies via IHC. As expected the positive control VZV infected human fibroblasts (HFs) readily stained for VZV IE63 (Figure 2F). IHC and immunofluorescence assay (IFA) was also performed using an antibody specific for the VZV glycoprotein E-glycoprotein I complex and VZV glycoprotein E, respectively and no VZV antigen expression was detected in either of the PHN ganglia (data not shown). Thus VZV antigen expression was not observed in human ganglia during PHN.

### Characterization of Infiltrating Immune Cells in Human Ganglia *in vivo*

**FIGURE 3:**
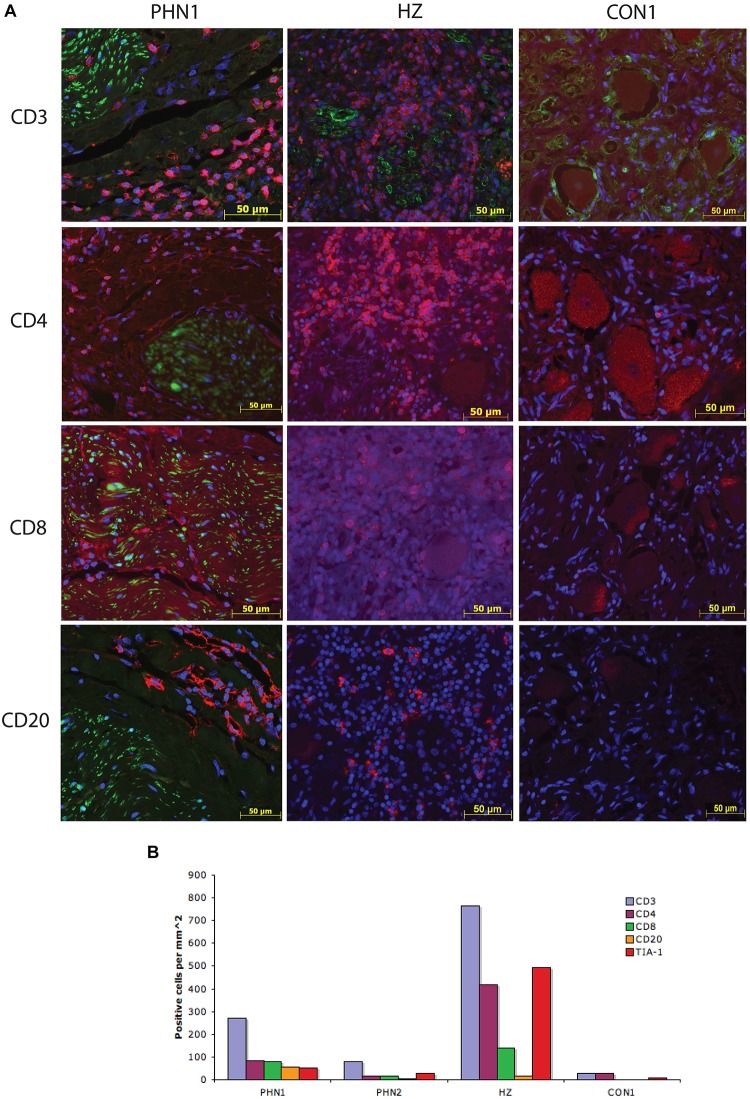
Characterization of the immune response within human ganglia during herpes zoster and post-herpetic neuralgia. **(A)** Representative images are shown for PHN1, HZ, and CON1. Primary antibodies used were specific for CD3, CD4, CD8, and CD20, and were detected using Alexa Fluor fluorescent conjugated antibodies (red). Sections were counterstained with DAPI (blue). All PHN sections were co-stained with all immune cell antibodies, HZ and CON1 with CD3 with an antibody specific for the satellite cell marker S100 (green). **(B)** The number of positive cells per square millimeter for each immune cell marker examined was determined for at least two independent stains and the average is shown.

Previous studies have shown the presence of immune like cells within PHN-affected ganglia material (Vafai et al., 1988; Watson et al., 1988b, 1991a), however, to date there has been no detailed characterization of the phenotype of these cells. To characterize the immune cells within the ganglia excised from a PHN-affected patient, and the ganglion innervating the site of reactivation of a HZ-affected patient, IFA staining for CD3, CD4, and CD8 to detect T cells, T helper and cytotoxic T cell subsets, and CD20 to detect B cells was performed. Cytotoxic T cells were further identified using the marker TIA-1. Isotype control antibodies were also utilized to establish the level of any non-specific background fluorescence. In both PHN ganglia and the HZ sample there was a large increase in the number of CD3^+^ T cells present when compared to the control samples but the number of CD3/CD4 lymphocytes was greatest in the HZ sample. Representative images are shown in Figure 3A, and frequencies of immune cells are shown in Figure 3B. Infiltration of CD3^+^ T cells was greatest in the HZ sample and these were observed throughout the neuronal regions of the ganglion. The PHN1 sample contained CD3^+^ T cells and CD20^+^ B cells in all regions of the ganglion, but with infiltrating cells spread throughout both neuronal and nerve bundle regions of the ganglion. PHN2 ganglion had an infiltration of CD3^+^ T and CD20^+^ B cells mainly restricted to the neuronal region. The PHN ganglia showed comparable frequencies of CD4^+^ and CD8^+^ T cells. This contrasted to that observed in HZ affected ganglia where there was a predominance of CD4^+^ T cells over CD8^+^ T cells (Figure 3B). Interestingly when staining for the cytotoxic marker TIA-1, TIA-1^+^ cells were readily observed in the HZ affected ganglia, but were less prominent in the PHN affected ganglia (Figure 3B). As expected there was limited staining for the various immune cell subsets in the control ganglia samples (Figures 3A,B).

Our data demonstrate a B cell and T cell immune infiltrate, with T cells displaying hallmarks of cytolytic T cells within PHN-affected human ganglia material obtained from a patient on long-term immunosuppressive therapy years following the resolution of the HZ associated rash.

## Discussion

Despite PHN having a significant impact on patient quality of life, there is a dearth of published studies investigating human ganglia during PHN. Notably only three publications have reported basic histological observations (Smith, 1978; Watson et al., 1988b, 1991b). Our study provides evidence that immune cells infiltrate ganglia during ongoing PHN and CD4^+^ and CD8^+^ T cells are a key feature of this process. Interestingly, despite the presence of immune cells a substantial viral DNA load was detected in these ganglia.

The PHN ganglia samples contained similar numbers of CD4^+^ and CD8^+^ T cells. Furthermore, there were TIA-1^+^ cells readily observed within these ganglia. This is in contrast to previous observations in ganglia from HZ-affected patients, which show a predominance of cytolytic CD4^+^ T cells during active HZ reactivation (Steain et al., 2014), and non-cytolytic CD8^+^ T cells in months following HZ reactivation (Gowrishankar et al., 2010). In the current study the number of TIA positive cells exceeded the number of CD8^+^ T cells. It is possible that this difference may be due to infiltrating cytolytic natural killer (NK) cells, as we have previously reported to be present in ganglia months after VZV reactivation (Gowrishankar et al., 2010).

One of the limitations of our study is the inherent difficulty in obtaining sensory ganglia from individuals who had HZ and suffered from PHN, and thus our sample size is small. This limits the extent to which we can make quantitative comparative analyses, although even with such small sample size, there appear to be differences in the nature of the immune infiltrate across the different patient groups. In addition, FFPE sections are notoriously difficult to unmask and detect antigens by IHC and IFA approaches. Staining for other immune cell markers such as NK cells, mast cells, macrophages, neutrophils and activation markers was not feasible due to technical constraints and the limited amount of ganglionic material available. Nonetheless, with the scarce ganglionic material available our data demonstrate a B cell and T cell immune infiltrate, with T cells displaying hallmarks of cytolytic T cells within PHN-affected human ganglia material obtained from a patient on long-term immunosuppressive therapy years following the resolution of the HZ associated rash. The PHN patient experienced subtotal relief of her neuralgia following both operations. The relief was stable over 2 years of clinical follow-up.

Post-herpetic neuralgia is the result of an as yet unidentified mechanism. In this study we have demonstrated persisting VZV DNA in ganglia during PHN (and years after HZ rash resolution). In the absence of detectable VZV antigen expression this may represent a latent pool of virus. In this respect, previous studies have reported in VZV latently infected ganglia a copy number of 258 of VZV DNA per 10^5^ cells (Pevenstein et al., 1999) and from 577 to 55,543 copies of VZV DNA per 10^5^ cells (Cohrs et al., 2000), with the latter quantity consistent with our estimate in PHN affected ganglia. It remains to be determined, however, whether reseeding of the latent pool following HZ may drive the immune response we detected in PHN, or whether there are small numbers of cells supporting undetected, persistent low level virus replication that may account for this immune infiltrate. This will be an important component of future studies to detect specific latent and lytic viral transcripts via *in situ* hybridization to further define the nature of viral genome persistence and its contribution to PHN. Irrespective of whether such viral genome persistence reflects true latency or perhaps a mixture of latent and low level productive and/or abortive infection, this study provides evidence of an ongoing immunological process that may contribute to the ongoing pain and pathology of PHN in this patient, years following HZ rash resolution.

## Materials and Methods

### Human Tissue Samples

All patient material was obtained in accordance with ethics guidelines of the University of Sydney and the Sydney Local Health District and informed consent of the donor was obtained where applicable. Post-mortem material were obtained from the Department of Forensic Medicine, Glebe, NSW, Australia, by following appropriate ethics approval from University of Sydney, Sydney Local Health District, and the coroner. Trigeminal and DRG fixed in 20% buffered formal were paraffin embedded. A limited number of 5 μm FFPE sections were obtained from each tissue block and mounted onto glass slides.

### DNA Extraction

DNA was extracted from FFPE tissue sections using the RecoverAll total nucleic acid isolation kit (Applied Biosystems, United States) as per manufacturer’s instructions.

### Primers

The human albumin-specific primer pair were as previously published (Douek et al., 2002). The VZV ORF28-specific primer pair sequences were forward CGAACACGTTCCCCATCAA and reverse CCCGGCTTTCTTAGTTTTGG, and the 6-carboxyfluorescein-linked (FAM) probe sequence was (FAM)-CCA GGTTTTAGTTGATACCA. HSV specific primers for UL42 were forward GCTTTGTGGTGCTGGTT and reverse CTGGT GCTGGACGACAC.

### Standard Curve for qRT-PCR

Standard curves were created using serial dilutions of a known amount of linearized plasmid constructs. Plasmid constructs consisted of pGEM-T Easy backbone (Promega, United States) containing small coding regions of human albumin or VZV ORF28 containing the region detected by the corresponding primer pairs.

### qRT-PCR Analyses

All samples were processed using a Rotorgene 6000 qRT-PCR machine (Qiagen, Australia). Data was analyzed using Rotorgene 6000 software (Qiagen, Australia). qRT-PCR for human albumin was performed using the SYBR Green system (Invitrogen, United States) as per manufacturer’s instructions. Cycling conditions were as follows: 50°C for 2 min, 95°C for 2 min, 45 cycles of 95°C for 10 s, 62°C for 15 s, 72°C for 20 s (acquiring fluorescence levels during this step). qRT-PCR for VZV ORF28 was performed using the Rotor-Gene Probe PCR kit (Qiagen, Australia). Cycling conditions were as follows: 95°C for 3 min, 50 cycles of 95°C for 3 s and 60°C for 10 s (acquiring fluorescence).

### Immunohistochemistry and Immunofluorescence Staining

Single and dual immunofluorescence staining was performed as previously described (Gowrishankar et al., 2010).

### Antibodies

The following primary antibodies and dilutions were used: mouse anti-human CD3 (Novocastra, Australia) (20 μg/mL), goat anti-human CD4 (R&D Systems, United States) (10 μg/mL), Rabbit anti-human CD8 (Abcam, United States) (2 μg/mL), mouse anti-human CD20 (Novocastra, Australia) (18 μg/mL), mouse anti-human T cell intracellular antigen 1 (TIA-1) (Beckman Coulter, Australia) (20 μg/mL), predilute rabbit anti-cow S100 (Dako, Denmark), rabbit anti-VZV IE63 polyclonal antibody (kindly provided by Prof Ravi Mahalingam, University of Colorado, Denver, CO, United States) and mouse anti-VZV gE:gI (Meridian Life Science, Saco, ME, United States). Isotype controls were mouse IgG_1_, mouse IgG_2__*a*_ (Invitrogen, United States), normal rabbit and normal goat IgG (R&D systems, United States), were diluted to match primary antibody concentrations. Secondary antibodies were AlexaFluor labeled antibodies (Molecular Probes, United States) at a dilution of 1:200.

### Imaging and Cell Counts

Imaging was performed using a Zeiss AxioPlan 2 upright microscope with an AxioCam camera (Zeiss, Australia). To perform cell counts 5–20 (depending on tissue size) random fields of view were captured and manual cell counting performed using the AxioVision image acquisition software. Counts from at least two independent stains were averaged. Tissue area for each field of view was calculated using the “Measure Outline” function of the AxioVisionLE microscope software. The data represents the average number of positive cells per square millimeter of regions of neuronal cell bodies and regions of nerve bundles.

## Data Availability

All datasets generated for this study are included in the manuscript and/or supplementary files.

## Ethics Statement

The studies involving human participants were reviewed and approved by The University of Sydney Ethics approval. Written informed consent for participation was not required for this study in accordance with the national legislation and the institutional requirements.

## Author Contributions

JS planned, performed the experiments, and analyzed the data. AA, BS, and MS planned the experiments and analyzed the data. MB and MR provided the samples. BS and AA received funding for the study. All authors contributed to writing of the manuscript.

## Conflict of Interest Statement

The authors declare that the research was conducted in the absence of any commercial or financial relationships that could be construed as a potential conflict of interest.
